# Maternal androgen excess inhibits fetal cardiomyocytes proliferation through RB-mediated cell cycle arrest and induces cardiac hypertrophy in adulthood

**DOI:** 10.1007/s40618-023-02178-1

**Published:** 2023-08-29

**Authors:** Y. Huo, W. Wang, J. Zhang, D. Xu, F. Bai, Y. Gui

**Affiliations:** 1 National Children’s Medical Center, Children’s Hospital of Fudan University, Fudan University, Shanghai, 201102 China; 2 National Health Commission (NHC) Key Laboratory of Neonatal Diseases, Fudan University, 399 Wanyuan Road, Minhang, Shanghai, 201102 China; 3 Cardiovascular Center, Children’s Hospital of Fudan University, Shanghai, 201102 China; 4 Institute of Pediatrics, Children’s Hospital of Fudan University, Shanghai, 201102 China; 5 Guangzhou Center for Disease Control and Prevention, Guangzhou, 510080 China

**Keywords:** DOHaD, Androgen excess, Cardiomyocytes’ proliferation, Cardiac function

## Abstract

**Purpose:**

Maternal hyperandrogenism during pregnancy is associated with adverse gestational outcomes and chronic non-communicable diseases in offspring. However, few studies are reported to demonstrate the association between maternal androgen excess and cardiac health in offspring. This study aimed to explore the relation between androgen exposure in utero and cardiac health of offspring in fetal and adult period. Its underlying mechanism is also illustrated in this research.

**Methods:**

Pregnant mice were injected with dihydrotestosterone (DHT) from gestational day (GD) 16.5 to GD18.5. On GD18.5, fetal heart tissue was collected for metabolite and morphological analysis. The hearts from adult offspring were also collected for morphological and qPCR analysis. H9c2 cells were treated with 75 μM androsterone. Immunofluorescence, flow cytometry, qPCR, and western blot were performed to observe cell proliferation and explore the underlying mechanism.

**Results:**

Intrauterine exposure to excessive androgen led to thinner ventricular wall, decreased number of cardiomyocytes in fetal offspring and caused cardiac hypertrophy, compromised cardiac function in adult offspring. The analysis of steroid hormone metabolites in fetal heart tissue by ultra performance liquid chromatography and tandem mass spectrometry showed that the content of androgen metabolite androsterone was significantly increased. Mechanistically, H9c2 cells treated with androsterone led to a significant decrease in phosphorylated retinoblastoma protein (pRB) and cell cycle-related protein including cyclin-dependent kinase 2 (CDK2), cyclin-dependent kinase 4 (CDK4), and cyclin D1 (CCND1) in cardiomyocytes. This resulted in cell cycle arrest at G1–S phase, which in turn inhibited cardiomyocyte proliferation.

**Conclusion:**

Taken together, our results indicate that in utero exposure to DHT, its metabolite androsterone could directly decrease cardiomyocytes proliferation through cell cycle arrest, which has a life-long-lasting effect on cardiac health. Our study highlights the importance of monitoring sex hormones in women during pregnancy and the follow-up of cardiac function in offspring with high risk of intrauterine androgen exposure.

## Introduction

Developmental Origins of Health and Disease (DOHaD) theory emphasizes that exposure to adverse factors, such as maternal malnutrition or overnutrition, or adverse endocrine environment during the embryonic, fetal, neonatal, and early childhood periods, will have a profound impact on disease susceptibility later in life [1–4]. So far, many epidemiological and animal research has supported the DOHaD and explored the underlying mechanisms [5–9]. The DOHaD theory provides a new perspective for the occurrence of chronic non-communicable diseases in adulthood, including cardiovascular diseases, and endocrine and metabolic diseases.

Polycystic ovary syndrome (PCOS) is a heterogeneous disease characterized by a combination of symptoms and signs of androgen excess and ovarian dysfunction [10, 11]. It is the most common endocrine disorder in women of reproductive age, with an incidence of 5–15% [10, 11]. Androgen excess is one of the most characteristic manifestations of women with PCOS [11]. Multiple reproductive and metabolic disorders in PCOS are related to hyperandrogenism, which is an important process of the occurrence and development of the disease [11–14]. Women with PCOS show hyperandrogenism in non-pregnancy, which would persist into pregnancy [15–17]. The placenta of women with PCOS undergoes morphological and structural changes, such as infarction, calcification, and increased villus space [18]. The newborns of pregnant women with PCOS also have lipid metabolism disorders manifested by increased cholesterol in the cord blood [18, 19]. These may be associated with adverse pregnancy outcomes for mothers with PCOS [20].

The incidence of cardiovascular events was increased in patients treated with androgen replacement therapy [21, 22]. The increased level of 5α-reductase in heart tissue of patients with heart failure suggested that dihydrotestosterone was accumulated in heart tissue [23]. Anti-androgen therapy can alleviate myocardial hypertrophy and left-ventricular dysfunction [23, 24]. All these phenomena suggest that androgens play a very important role in the occurrence and development of cardiovascular diseases. However, whether the elevated level of maternal circulating androgen during pregnancy affects the changes of fetal cardiac morphology and structure, thereby increasing the risk of cardiovascular disease in adulthood is rarely reported.

During the embryonic period, cardiomyocytes exhibit impressive proliferative capacity, which decreases dramatically after birth [25]. Cardiomyocyte proliferation is essential for the morphogenesis, development, and maintenance of normal function of the heart [26, 27]. The rapid turnover of mammalian myocardial cell cycle is the fundamental reason for the different growth pattern of cardiomyocytes before and after birth [28]. Retinoblastoma (RB) plays a pivotal role in regulating cell cycle progression. Cyclin-dependent kinase 2 (CDK2) and cyclin-dependent kinase 4 (CDK4) regulate RB hyper-phosphorylation status and prevent RB from binding with E2F transcription factors, thereby promoting cell cycle progression [28]. Accumulating evidence has demonstrated that RB is related to the regulation of cardiomyocyte proliferation, cardiomyopathy, and ventricular remodeling in heart failure [29–31]. RB also plays an important role in atrial fibrillation caused by diabetes [32]. However, it remains unclear whether RB plays a role in the changes of cardiac morphology and function in offspring caused by a detrimental maternal environment due to androgen exposure.

Therefore, we hypothesize that maternal androgen excess in late pregnancy causes the growth inhibition of fetal cardiomyocytes, which would lead to impaired cardiac structure and function in adulthood. To verify the hypothesis, we used an animal model of intrauterine hyperandrogenic environment as mentioned in the literature. Cardiomyocytes were also treated with androsterone to investigate the underlying mechanism of the limited cardiomyocyte proliferation induced by excessive androgen exposure.

## Materials and methods

### Animal model

**Fig. 1:**
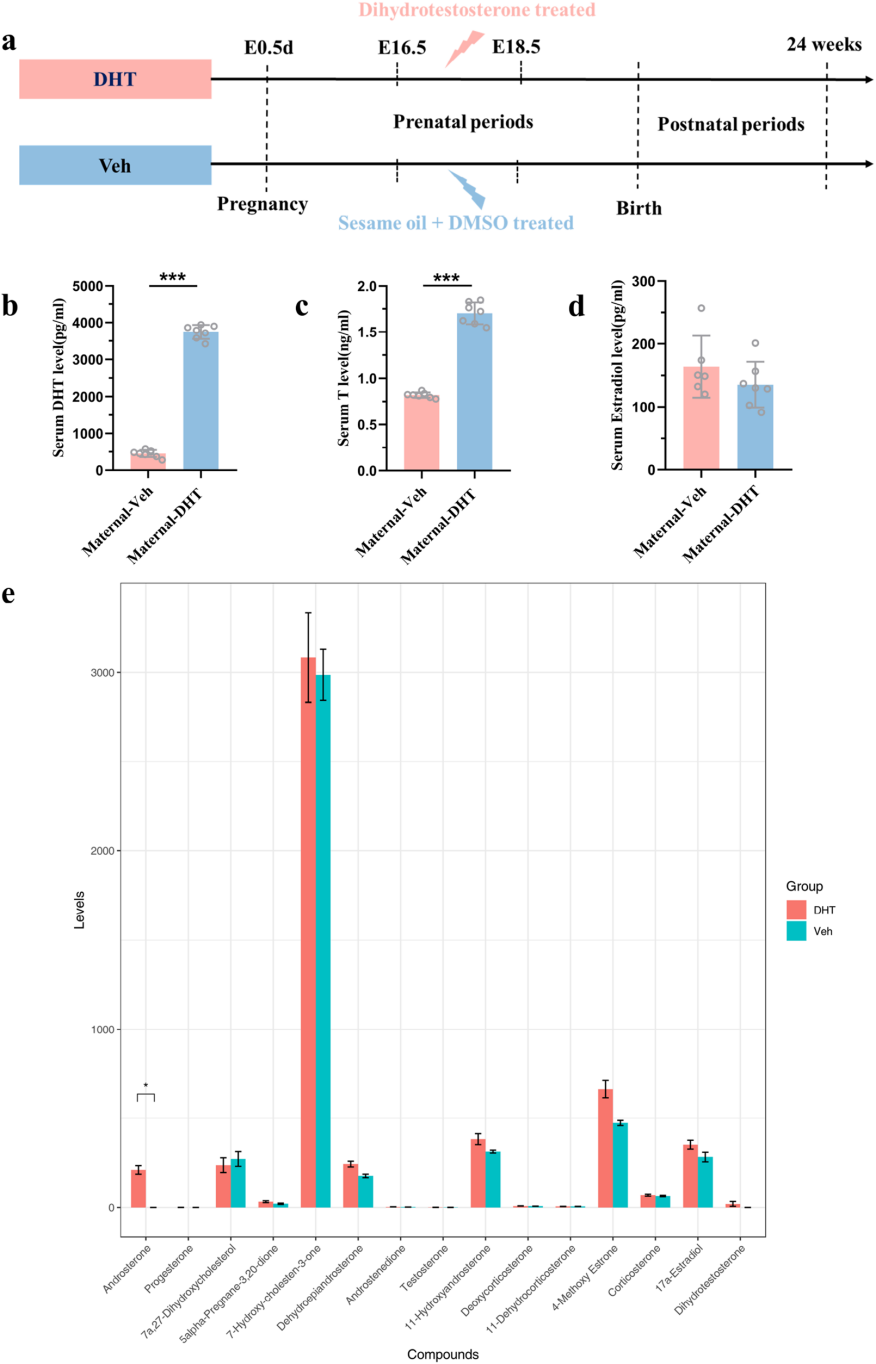
Maternal serum DHT, T, estradiol, and the content of steroid hormones in fetal cardiac tissue. **a** Schematic of the protocol. **b** Maternal circulating DHT levels, **c** T levels, and **d** estradiol levels on GD 18.5 (*n* = 6–8 animals per group). **e** Cardiac-tissue steroid hormones contents (*n* = 12 animals per group). Data were analyzed with two-tailed Student’s *t* test, *p < 0.05, ***p < 0.001. Bars represent mean values, and error bars represent standard deviation. DHT, prenatal androgen exposure; Veh, vehicle exposure

Nine-week-old female mice (C57BL/6JNifdc) were purchased from Vital River (Zhejiang, China), housed four per cage, and maintained at 22 °C with 55–65% humidity and a 12-h light/dark cycle. They are free to drink water and eat food from the cage. The protocol of mouse model establishment is shown in Fig. 1a. Females and males were mated in a 2:1 manner after a week of adaptive feeding. Females were examined daily for vaginal plugs, and a plug on the morning after mating was considered as gestational day (GD) 0.5. From GD16.5 to 18.5, the control and DHT-treated group were injected subcutaneously with 100 μL vehicle or 250 μg dihydrotestosterone (DHT) (Molnova, M20270) dissolved in a mixture of 1 μL DMSO (Solarbio, D8371) and 99 μL sesame oil (Molnova, M25057). After 2 h of injection on GD18.5, parts of dams were euthanized by isoflurane inhalation and subjected to a cesarean section. The fetuses and fetal hearts were quickly dissected and dried on the medical gauze to remove any remaining fetal membranes, weighed, snap frozen, and stored at − 80 °C for further analysis. Maternal serum was collected through eyeball extirpating and frozen at − 80 °C for the further measurement of DHT, testosterone (T), and estradiol. The others were delivered at term, weaned on day 21, and fed an in-house chow diet until postnatal 24 weeks. Cardiac structure and function of the offspring in the two groups were assessed. All animal experiments were approved by the Ethics Committee of Children's Hospital of Fudan University [No. (2022) 384].

### Serum levels of steroid hormones

Enzyme-linked immunosorbent assay (ELISA) kits were used to measure the levels of maternal serum DHT (LDN, AA E-1900), T (LDN, AR E-8000), and estradiol (LDN, FR E-2000) on GD18.5. All assays were performed according to the manufacturer’s instructions.

### The levels of steroid hormones in fetal heart

Fetal cardiac tissue was mixed with precooled 70% methanol 500 μL, vortexed for 3 min, and centrifuged 12,000 r/min for 10 min at 4 °C. Then, 300 μL of supernatant was aspirated and stored at − 20 °C for 30 min, followed by centrifuging 12,000 r/min for 10 min at 4 °C. Finally, 200 μL of the supernatant was used for further analysis. Different concentrations of standard solutions were prepared and standard curves were drawn for quantitative analysis of the metabolites. Data collection and analysis of hormones and the metabolites was based on ultra performance liquid chromatography (UPLC) (ExionLC™ AD, https://sciex.com.cn/) and Tandem Mass Spectrometry (MS/MS) (QTRAP^®^ 6500+, https://sciex.com.cn/) by MetWare (http://www.metware.cn/).

### Histological analysis

Fetal hearts on GD18.5 and adult hearts on 24 weeks were fixed with 4% paraformaldehyde (Servicebio, G1101), paraffin-embedded, processed, and sectioned at 4 μm. Hematoxylin and eosin (H&E) reagent (Servicebio, G1003), FITC-conjugated wheat germ agglutinin (WGA) (sigma, L4895), and Masson (Servicebio, G1006) were used to evaluate the cardiomyocytes cross-sectional areas and the extent of myocardial fibrosis, respectively. More than 200 randomly cardiomyocytes were selected to calculate the cross-sectional area for one slide. Fibrosis area was assessed by randomly selecting at least three fields from one slide. Cell size and fibrotic area were measured by ImageJ.

For immunohistochemistry, the slides were deparaffinized, rehydrated, and antigen retrieval. Then, the slides were immersed in 3% H_2_O_2_ (Servicebio, G0115) and incubated at room temperature for 20 min, followed by 3% BSA (Bovine Serum Albumin) (Servicebio, GC305010) blocking for 30 min at room temperature. The sections were incubated with Ki67 reagent (Servicebio, GB111141) at 4 °C overnight. After three washes, the sections were incubated with Goat anti-rabbit with HRP (Servicebio, G1213). Finally, the positive cells detected by 3,3′-diaminobenzidine (DAB) (Servicebio, G1212) were shown brown. The ratio of positive cells reflecting the proliferation of cardiomyocytes was measured by ImageJ.

### TUNEL staining

Cardiomyocyte apoptosis was demonstrated by terminal deoxynucleotidyl transferase-mediated deoxyuridine triphosphate nick-end labeling (TUNEL) staining. It was performed using the DAB (SA-HRP) Tunel Cell Apoptosis Detection Kit (Servicebio, G1507-100T) following the manufacturer’s instructions. The positive apoptotic nuclei shown by DAB were brown, while the normal nuclei stained with hematoxylin were blue.

### Transthoracic echocardiography

On 24 weeks of age, cardiac function was non-invasively assessed by transthoracic echocardiography using Vevo 2100 (VisualSonics, Toronto, Ontario, Canada). The mice were anaesthetized by isoflurane (1–1.5%) inhalation. Left-ventricular posterior wall thickness (LVPW), left-ventricular internal dimension (LVID), and interventricular septum (IVS) at both end-systole and end-diastole were measured. The LV mass and left-ventricular ejection fraction (LVEF) were calculated by VisualSonics software.

### Cell culture

Rat cardiomyoblast cells (H9c2 cells) was purchased from Pricella (Wuhan, China) and incubated with high-glucose DMEM (Pricella, PM150210A) supplemented with 10% fetal bovine serum (Gibco, 10100147) in a humidified atmosphere at 37 °C with 5% CO_2_. Cells were lysed for RNA, protein extraction, and immunofluorescence staining.

### CCK-8 assay

H9c2 cells treated with different concentrations of androsterone were seeded in 96-well culture plates (5000/well) and incubated for different period. The old solution was discarded and a mixture of 10 μL CCK-8 solution (Beyome, C0038) and 90 μL DMEM medium was added to every well at 12 h, 24 h, 48 h, and 72 h, respectively. The OD value at 450 nm was measured by the microplate reader, and the cell viability was calculated by the formula: cell viability (%) = (OD_DHT_ − OD_blank_)/(OD_control_ − OD_blank_) × 100. OD_control_: absorbance of wells with cells, CCK-8 solution but no androsterone. OD_DHT_: absorbance of wells with cells, CCK-8 solution and androsterone. OD_blank_: absorbance of wells with medium and CCK-8 solution without cells.

### Immunofluorescence staining

H9c2 cells were fixed with 4% paraformaldehyde, and then permeabilized and blocked in QuickBlock™ Blocking Buffer for Immunol Staining (Beyome, P0260). Next, the cell slides were incubated with Ki67 (Abcam, ab16667) at 4 °C overnight and secondary antibody Goat anti-Rabbit IgG-AlexaFluor 488 (Absin, abs20025) for 1 h. Finally, the cell slides were stained with DAPI (Servicebio, G1012) and observed with confocal fluorescence microscopy. The proportion of positive cells was used to reflect cell proliferation.

### RNA extraction and quantitative real-time PCR

The total RNA was extracted from adult hearts and H9c2 cells by Trizol (Invitrogen, 15596026) and reverse transcribed into cDNA in a total volume of 20 μl using Evo M-MLV RT Mix Kit with gDNA Clean for qPCR (Accurate Biotechnology, AG11728), according to the manufacturer’s instruction. Quantitative real-time PCR (qRT-PCR) was performed to quantify the relative mRNA levels using a Light Cycler 480 real-time PCR system (Roche, Switzerland) and SYBR Green Pro Taq HS Premix kit (Accurate Biotechnology, AG11701); *β-actin* and *Gapdh* was used as the internal control, and the relative mRNA levels were calculated using 2^− ΔΔCT^ method. All primers used in the study are described in Table 1.

**Table 1:** Sequence of the primers for real-time qPCR used in this study

	Forward primer	Reverse primer
Rat		
*CCNE1*	TTCTTCTGGACTGGCTGATG	TGTTGTGATGCCATGTAAC
*CCNE2*	GGGAAACATTTTATCTTGCACA	CTGCAAGCACCATCAGTGAC
*CCNB1*	ATCGGTTCATGCAGGACAGT	TGGAGGGTACATCTCCTCGT
*CDK2*	AAGATCGGAGAGGGCACGTA CGGAGTGGTG	AGGCTCTTGCTAGTCCAAAG TCTGCCAACT
*AURKB*	AGTGCTCTGCTATGAACTGATG	ACCTTGACAATCCGACGATATG
*β-actin*	CGCGAGTACAACCTTCTTGC	CGCAGCGATATCGTCATCCAT
Mouse		
*Nppa*	ATTGACAGGATTGGAGCCCAGAGT	TGACACACCACAAGGGCTTAGGAT
*Nppb*	CTCAAGCTGCTTTGGGCACAAGAT	AGCCAGGAGGTCTTCCTACAACAA
*Myh6*	ATAAAGGGGCTGGAGCACTG	AGGAACAGGCAGGAAGAGGA
*Myh7*	TCCTGCTGTTTCCTTACTTGC	GCCTTGGATTCTCAAACGTGTC
*Acta1*	GAAGCCTCACTTCCTACCCT	CGTGTGGCTCAGTAGGAGAG
*Gata4*	GAAGACACCCCAATCTCGATATG	GGCATTGCACAGGTA GTGTCC
*Col1a1*	GATGACGTGCAATGCAATGAA	CCCTCGACTCCTACATCTTCTGA
*Col3a1*	GACCAAAAGGTGATGCTGGACAG	CAAGACCTCGTGCTCCAGTTAG
*Tgfβ1*	GACCGCAACAACGCCATCTA	GGCGTATCAGTGGGGGTCAG
*Gapdh*	AGGTCGGTGTGAACGGATTTG	GGGGTCGTTGATGGCAA

### Western blotting

Protein was extracted from H9c2 cells using RIPA lysis buffer (Sangon Biotech, C510006) with a 1% proteinase and phosphatase inhibitor mixture (Thermo Scientific, 78,441). Protein concentrations were determined using a BCA Protein Assay Kit (Beyotime, P0012S). Equal amounts of proteins were separated by 8–12% SDS-PAGE gels and transferred to 0.22 µm polyvinylidene fluoride membranes (cytiva, 10600021). Then, the membranes were blocked in Quick blocking buffer (Beyotime, P0252) for 1 h and incubated with primary antibody at 4 °C overnight, followed by the corresponding HRP-conjugated secondary antibodies. The bands were visualized using an ECL kit (Biosharp, BL520A) and imaged with a ChemiDoc™ XRS+ Imager (Bio-Rad). ImageJ was used to quantify the protein bands, and all relative protein levels were normalized to *β-actin*. The antibodies were used as Table 2.

**Table 2:** Antibodies used in this study

Antibody	Host species	Catalogue number	Manufacturer	Final concentration
*CCNB1*	Rabbit	AF6168	Affinity	1:1000
*CCND1*	Rabbit	#55506	CST	1:1000
*CDK2*	Rabbit	#18048	CST	1:1000
*CDK4*	Rabbit	DF6102	Affinity	1:1000
*RB*	Rabbit	10048–2-lg	Proteintech	1:5000
*p-RB*	Rabbit	#8516	CST	1:1000
*β-actin*	Mouse	T0022	Affinity	1:5000

### Flow cytometry for cell cycle analysis

Cell Cycle and Apoptosis Analysis Kit (Beyotime, C1052) was used to analyze the cell cycle with a flow cytometer (BD FACSCelesta, San Jose, CA, USA) following the manufacturer’s protocol. H9c2 cells were cultured in 6-well plates (2 × 10^5^ cells/well) and treated with androsterone for 36 h. The cells were digested with ethylenediaminetetraacetic acid (EDTA)-free trypsin and stained with PI. The data were analyzed using FlowJo V10 software (Tree Star, USA).

### Statistical analysis

All values are presented as mean ± standard deviation, and the statistical analysis was performed by Graphpad Prism 8.0. All independent variables were tested for normality and homogeneity of variance. Statistical analysis between two groups was performed by the unpaired Student's *t* test, and one-way ANOVA was used for comparison between multiple groups. *p* value less than 0.05 was considered as statistically significant.

## Results

### Androsterone, a metabolite of androgens, was increased in the maternal DHT-treated fetal hearts

The prenatally androgenized offspring were established as shown in Fig. 1a. Pregnant dams were treated with low-dose DHT on GD16.5–18.5. We use DHT instead of T to avoid the potential effects of T aromatization, which would produce estradiol [35]. The pregnant mice in Maternal-DHT group had a significant increase in circulating T and DHT levels on gestational 18.5d compared with Maternal-Veh group (Fig. 1b, c). There was no significance in Estradiol levels between two groups (Fig. 1d). We also tested steroid hormones and the metabolites in cardiac tissue of fetus on GD18.5. The results are shown in Fig. 1e. The fold change ≥ 1.5 or fold change ≤ 0.67 and *p* < 0.05 were used as the screening criteria of differential metabolites. A total of 15 metabolites were detected. Only the level of androsterone, a metabolite of androgens, was significantly increased in the maternal DHT-treated fetal hearts compared with maternal Veh-treated group. There was no significance in other metabolites between the two groups.

### Maternal androgen excess induced limited cardiomyocytes’ proliferation in fetal period

**Fig. 2:**
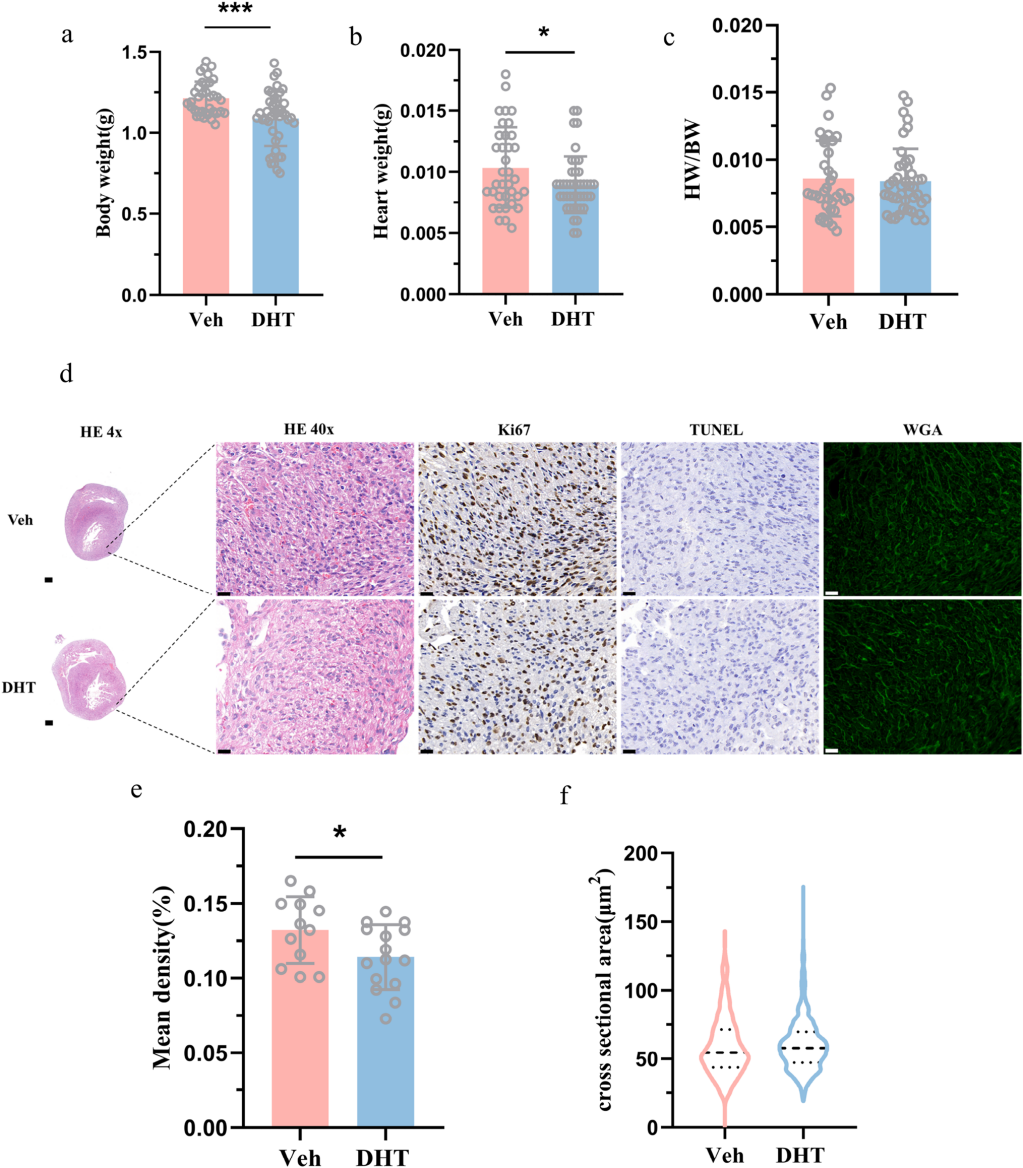
Cardiac morphological changes of fetus exposed to hyperandrogenic environment. **a** Body weight, **b** heart weight, and **c** HW/BW ratio in fetus of maternal-Veh and maternal-DHT group (*n* = 36–43 animals per group). **d** Fetal heart sections stained with H&E, Ki67, TUNEL, and WGA (*n* = 12–15 per group). **e** Quantification of Ki67 density. **f** Violin plots showing the distribution of relative sizes of cardiomyocytes. Data are shown as mean ± standard deviation, analyzed by two-tailed Student’s *t* test. *p < 0.05, ***p < 0.001. Scale bar: 200 μm; Scale bar: 20 μm

The fetal body weight and heart weight were significantly decreased in maternal DHT-treated group (Fig. 2a, b). However, there was no difference in the ratio of heart weight and body weight between the two groups (Fig. 2c). To identify the reason of the reduction in heart weight, we performed H&E staining of the tissues as shown in Fig. 2d. The maternal DHT-treated fetal heart developed normally but exhibited a thinner left-ventricular (LV) wall and a larger LV chamber. High magnification images of LV free wall in maternal DHT-treated group with H&E staining showed dysregulation of cardiomyocytes and reduced cellular density resembling non-compaction phenotype (Fig. 2d). The WGA staining showed no difference in the cross-sectional area of the fetal hearts between the two groups (Fig. 2d, f). To clarify the cause of reduction in cardiomyocytes, we performed Ki67 and TUNEL staining to assess the proliferation and apoptosis of cardiomyocytes, respectively. As shown in Fig. 2d, the proliferation of myocardial cells was markedly decreased, but there was no difference in cardiomyocytes apoptosis (Fig. 2d, e). These results suggested that reduced cellular density may be due to the decreased proliferation of cardiomyocytes rather than cell apoptosis.

### Lower cellular density in fetal heart induced cardiac hypertrophy and fibrosis in adulthood

**Fig. 3:**
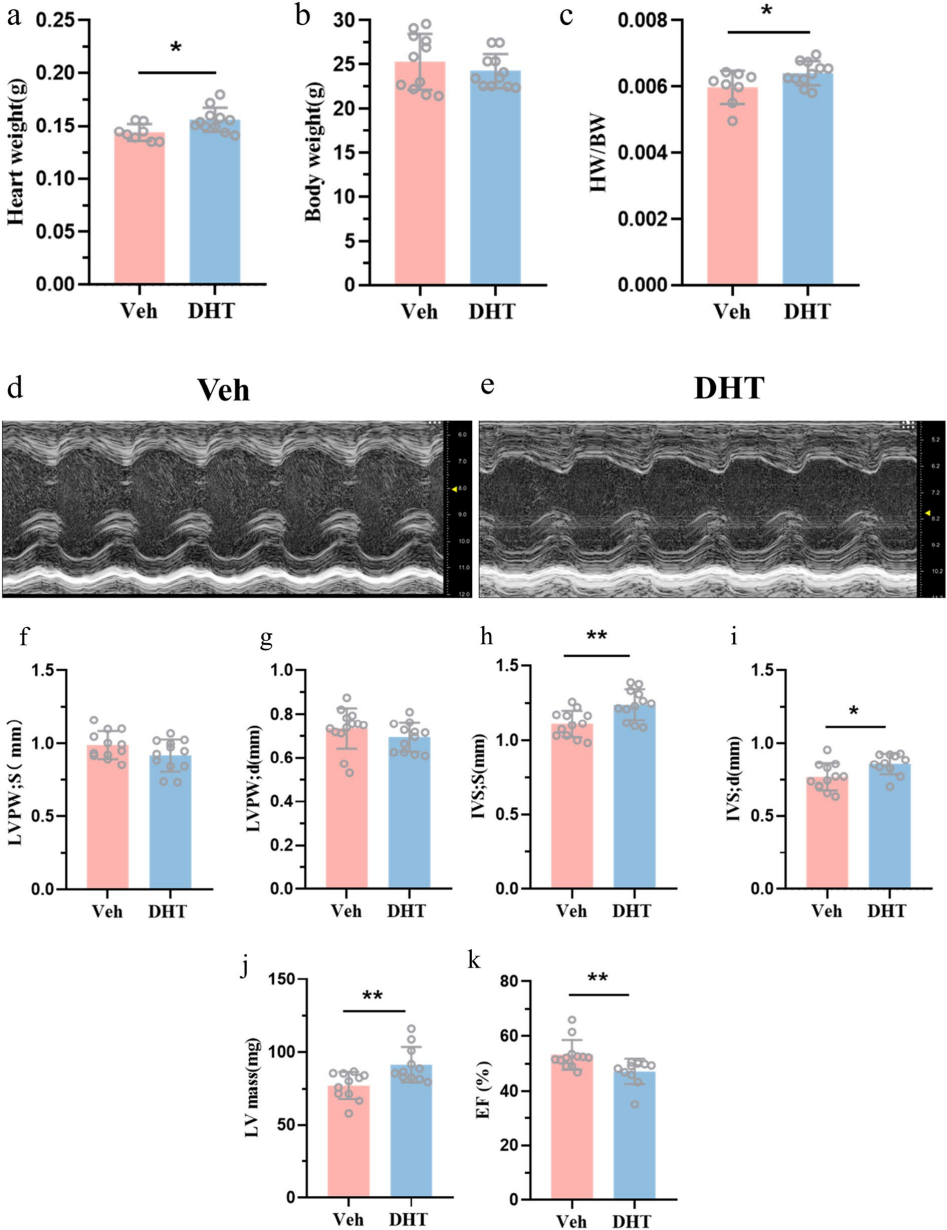
Cardiac function of adult offspring at 24 weeks exposed to hyperandrogenic environment. **a** Heart weight, **b** body weight, and **c** the ratio of heart weight and body weight of adult offspring at 24 w in control and prenatally androgenized group (*n* = 8–11 animals per group). **d**, **e** Assessment on cardiac function using M-mode echocardiography. **f–k** Measurement of echocardiographic parameters in adult offspring (*n* = 11–13 animals per group). Data represent the mean ± standard deviation, and were analyzed by unpaired two-tailed Student’s *t* test. *p < 0.05, **p < 0.01

To investigate whether prenatal suppression of cardiomyocytes’ proliferation increases the risk of cardiovascular disease in adulthood, we observed changes in cardiac structure and function in 24 w offspring. Body weight at 24 w showed no difference between maternal DHT-treated group and Veh-treated group (Fig. 3b). While, the heart weight and the ratio of heart and body weight were significantly higher in maternal DHT-treated group (Fig. 3a, c). The cardiac structural and functional changes were assessed with echocardiography (Fig. 3d, e). We found no difference in LVPWs (s: end-systole) and LVPWd (d: end-diastole) between the two groups (Fig. 3f, g), while the left-ventricular mass, IVSs, and IVSd of the 24 weeks offspring in maternal DHT-treated group were significantly greater than those of Veh-treated group (Fig. 3h–j). The LVEF (%) in maternal DHT-treated group was remarkably decreased (Fig. 3k).

**Fig. 4:**
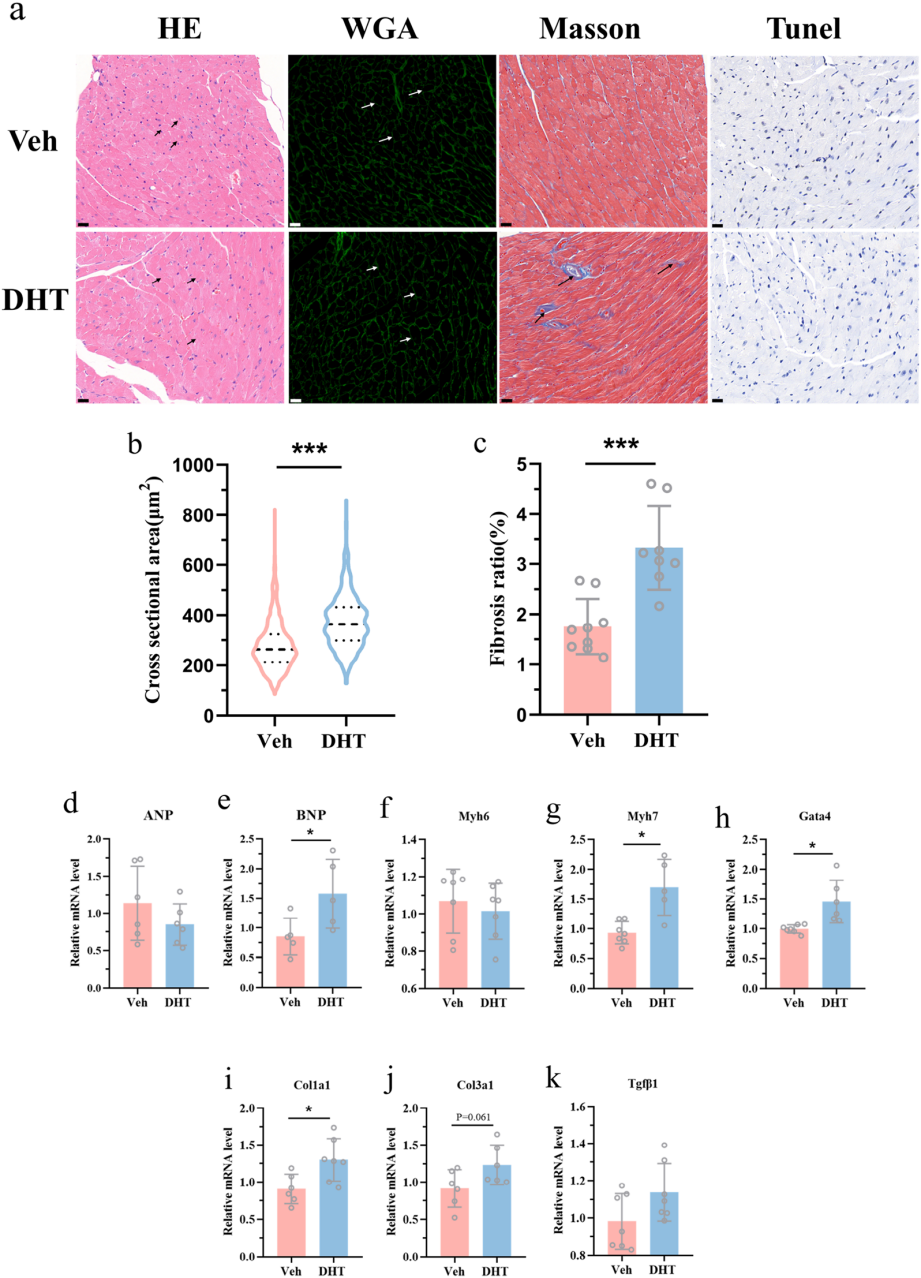
Changes in myocardial cell morphology and related gene expression. **a** Representative images of cardiac sections stained with H&E, WGA, Masson, and TUNEL. Scale bar: 20 μm. **b**, **c** Measurement of cardiomyocytes cross-sectional areas and fibrosis ratio of 24 weeks offspring in control and prenatally androgenized group (*n* = 8–9 animals per group). **d–k** Changes in hypertrophy-related genes and fibrosis-related genes expression (*n* = 5–7/group). The data are shown as mean ± standard deviation, analyzed by two-tailed t test. *p < 0.05, ***p < 0.001

To further investigate the cause of ventricular septum thickening and cardiac function decline, the changes of cardiomyocytes were observed by histomorphological staining. H&E and WGA staining demonstrated that the cross-sectional area of cardiomyocytes was significantly increased (Fig. 4a, b). Masson staining revealed the increased myocardial cell fibrosis in maternal DHT-treated group (Fig. 4a, c). However, there was no difference in myocardial apoptosis between the two groups (Fig. 4a). These results suggested that increased septal thickness and abnormal function were due to cardiomyocyte hypertrophy and fibrosis rather than apoptosis. Meanwhile, these results were also supported by mRNA levels. We examined cardiac hypertrophy-related genes and found that the mRNA levels of *BNP* (Natriuretic Peptide B),* Myh7* (Myosin Heavy Chain 7), and *Gata4* (GATA Binding Protein 4) were significantly up-regulated in maternal DHT-treated group (Fig. 4d–h). We also assessed the expression of genes related to fibrosis. *Col1a1* (Collagen Type I Alpha 1 Chain) was significantly up-regulated. *Col3a1* (Collagen Type III Alpha 1 Chain) and *Tgfβ1* (Transforming Growth Factor Beta 1) were also in an upward trend (Fig. 4i–k). Generally, the inhibition of fetal cardiomyocytes proliferation caused by maternal androgen excess led to abnormal cardiac structure and function in adulthood.

### Androsterone treatment inhibited the proliferation of H9c2 cells

**Fig. 5:**
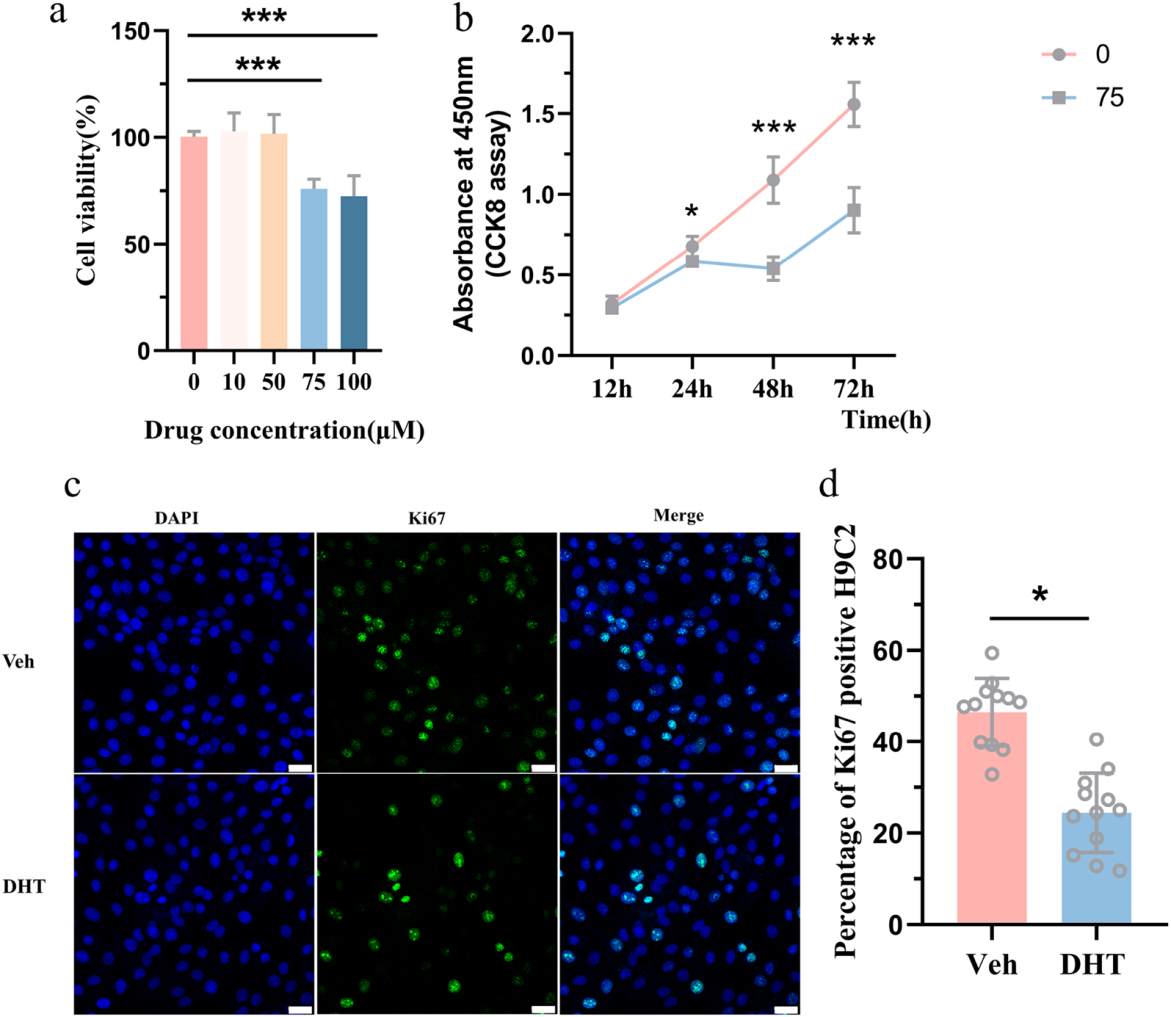
H9c2 cells’ proliferation with androsterone treatment. **a** Changes in H9c2 cells’ viability treated with different concentrations of androsterone for 24 h, detected by CCK8 assay. **b** Graph on H9c2 cells’ viability treated with 75 μM androsterone changing over time. **c** H9c2 cells’ immunofluorescence stained with Ki67 to reflect cell proliferation under 75 μM androsterone treatment, with green and blue indicating Ki67 and DAPI, respectively. Scale bar: 25 μm. **d** Quantification of Ki67 density. Data are shown as mean ± standard deviation, analyzed by two-tailed Student’s *t* test. *p < 0.05

Based on the above observation, we want to clarify the underlying mechanism of inhibited cardiomyocytes proliferation. Thus, we constructed a cell model of androgen exposure. Due to the remarkably increased androsterone levels in maternal DHT-treated dams in myocardial tissue, we treated H9c2 cells with different concentrations of androsterone for 24 h. 75 μM and 100 μM androsterone treatment markedly decreased H9c2 cells’ viability (Fig. 5a). The suppression effects were more obvious over time (Fig. 5b). Meanwhile, we use Ki67 immunofluorescence staining to observe cell proliferation. Androsterone treatment significantly decreased H9c2 cells’ proliferation (Fig. 5c, d).

### Altered RB signaling pathway caused cell cycle arrest

**Fig. 6:**
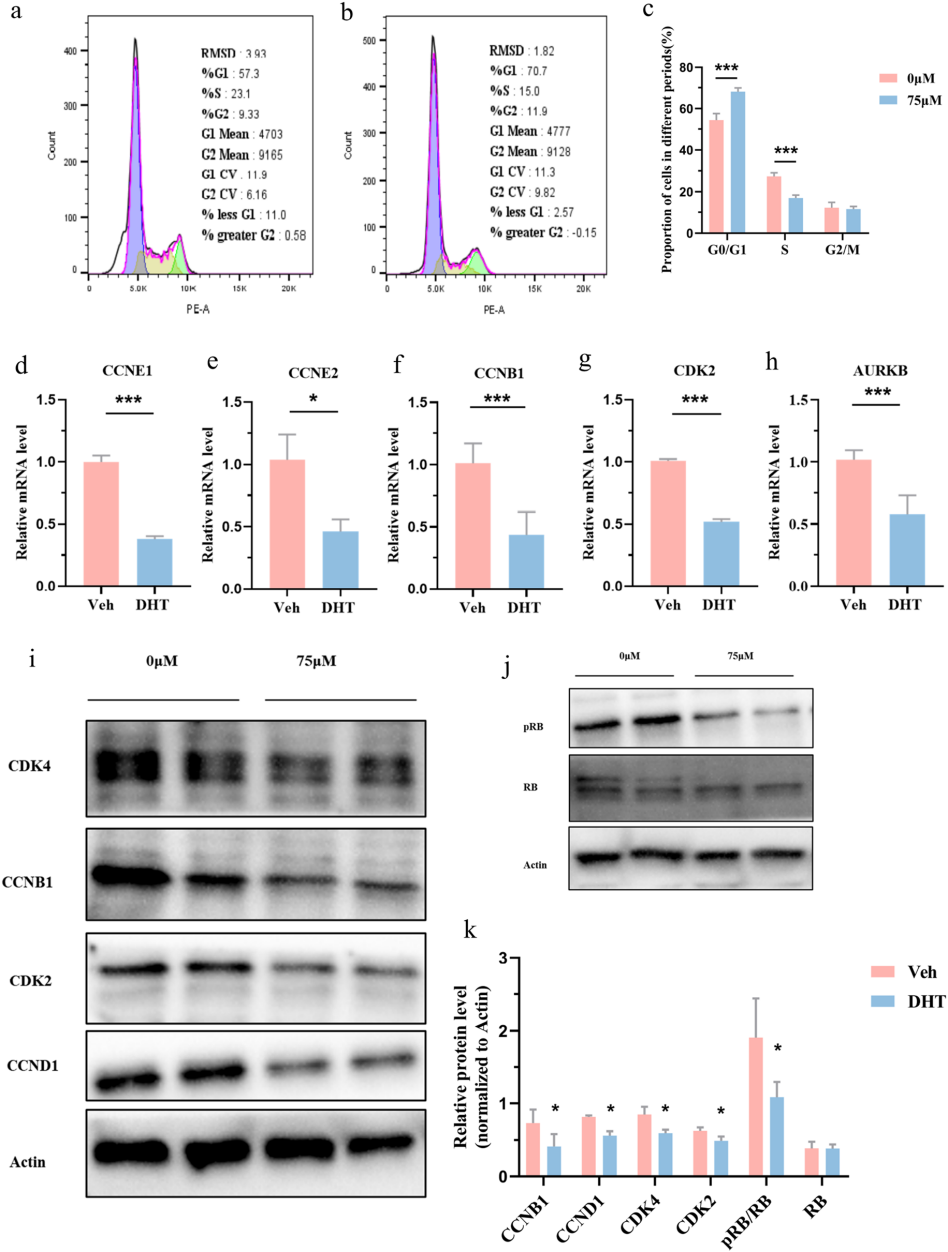
Inhibition of cell proliferation was caused by cell arrest in G1–S stage through RB signaling pathway. **a**, **b** H9c2 cells with control and 75 μM androsterone treatment, respectively, for 36 h for analyzing cell cycle profiles by flow cytometry. **c** Analysis by the FlowJo to display the cell cycle distribution. **d**–**i** Changes in cell cycle-related genes expression and protein levels treated with control and 75 μM androsterone in H9c2 cells. **j** Changes in the important regulator of cell cycle RB protein levels. **k** Corresponding quantification of CDK4, CCNB1, CDK2, CCND1, pRB/RB, and RB in control and H9c2 cells treated with 75 μM androsterone. The data are shown as mean ± standard deviation, analyzed by two-tailed *t* test. *p < 0.05, **p < 0.01, ***p < 0.001

To explore the cause of limited cell proliferation caused by androsterone treatment, cell cycle was analyzed by flow cytometry. The results showed that cells in G1 phase were significantly increased, while cells in S phase were declined in androsterone-treated group (Fig. 6a–c). We also examined the changes in gene expression of cyclins and cyclin-dependent kinase. qPCR analysis demonstrated that the mRNA levels of CCNB1 (Cyclin B1), CCNE1 (Cyclin E1), CCNE2 (Cyclin E2), CDK2, and AURKB (Aurora Kinase B) were significantly decreased in androsterone-treated group (Fig. 6d–h). Western blotting also revealed that cyclins and cyclin-dependent kinase, including CCNB1, CCND1 (Cyclin D1), CDK2, and CDK4, had a remarkably reduction in androsterone-treated group (Fig. 6i, k). All these results suggested that the inhibition of cell proliferation was caused by cell cycle G1–S arrest. We speculated that the cell cycle arrest caused by androsterone treatment might be associated with the hypophosphorylation of RB protein, which is a key regulatory molecule of the cell cycle. Subsequent the results revealed that phosphorylated RB protein was significantly decreased in androsterone-treated group (Fig. 6j, k). In conclusion, the suppression of cell proliferation resulted from androsterone treatment is caused by the alternation of the phosphorylation status of RB protein, which led to cell cycle arrest.

## Discussion

Many studies have shown that elevated androgen levels during pregnancy may be associated with an increased risk of cardiovascular disease in offspring [24, 33–35]. However, it is still unknown whether excessive maternal androgen levels could directly damage fetal cardiac structure and function. Unlike other reports, we are the first to analyze the steroid metabolites in fetal heart tissue exposed to intrauterine hyperandrogenic environment. Our results showed that the level of androsterone in the fetal heart was markedly increased in the DHT-treated group, while the levels of testosterone and dihydrotestosterone did not change significantly. This might be a protective mechanism to avoid fetal exposure to excessive androgen environment through maternal, placental, and/or fetal metabolism [36]. These results are consistent with a previous study suggesting prenatal androgen exposure increased offspring levels of androsterone rather than testosterone or dihydrotestosterone [37].

Some literature has proposed that maternal androgen excess could induce cardiac hypertrophy and compromised cardiac function in adult period [24, 35]. However, whether the abnormal structure and function of cardiac in adulthood has a fetal basis is unknown. Our results showed that elevated androgen levels led to a decrease in fetal heart weight and body weight. However, the ratio of heart weight to body weight did not change significantly between the two groups. These suggest that intrauterine growth restriction occurred and the small heart might be appropriate for the body size. However, even if the heart weight and body size are compatible, it still reflects the aberrant cardiomyocytes during development, which may contribute to an increased risk of cardiovascular disease later in life [38]. Indeed, this hypothesis is also supported by our subsequent observations of embryonic and adult phenotypes.

The heart is a terminally differentiated organ. The cardiomyocytes proliferate rapidly during the fetal period and decline sharply during the perinatal period [39, 40]. This is very important for the development and maturation of heart [41, 42]. We found that fetal heart showed a thinner ventricular wall and larger ventricular chamber, which may be due to the inhibition of fetal cardiac cell proliferation. The DOHaD suggests that chronic non-communicable diseases in adulthood may attribute to adverse factors exposure during pregnancy [43]. Therefore, we followed up the cardiac structure and function of the offspring in adulthood. Adult offspring exposed to intrauterine hyperandrogenism developed cardiac hypertrophy and fibrosis, resulting in compromised cardiac function, which was consistent with the previous study [24, 35]. Intrauterine exposure to high androgen environment decreases cardiomyocyte proliferation in fetal period and results in cardiomyocyte hypertrophy in adulthood. This seemingly paradoxical result may be due to the inhibition of cardiac proliferation in the embryonic period leading to a decrease in the absolute number of cardiomyocytes, while the proliferation ability of cardiomyocytes declines sharply from birth [25]. There was no difference in body weight of 24w offspring, indicating that there may be a phenomenon of catch-up growth [24]. Cardiomyocytes can meet the growth requirements through compensatory hypertrophy [44]. Therefore, all these phenomena suggest that the effects of in utero androgen exposure on the offspring’s heart are mild but long-lasting throughout life. It reminds us to pay attention to the changes of sex hormone levels during pregnancy, especially in patients with PCOS and other diseases that can cause androgen elevation before and during pregnancy. On the other hand, it also reminds us to follow up the cardiac structure and function of offspring exposed to hyperandrogenism during pregnancy.

The poor prognosis of many cardiovascular diseases is due to the limited regenerative capacity of cardiomyocytes, which may be caused by cell cycle arrest of cardiomyocytes after birth [45, 46]. The normal progression and exit of the cell cycle are important for the development and maturation of cardiomyocytes [39, 47]. Cell proliferation is controlled by the activation of two classes known as cyclins and cyclin-dependent kinases [28]. The two families of proteins coordinate with each other and form complexes, prior to DNA synthesis and cell division [28]. CDK2/CDK4 and cyclin D are essential for normal cardiac development [39]. Loss of both cyclin D and CDK2/CDK4 results in cardiac defects and embryonic lethality [48, 49]. The heart showed reduced volume, thinner ventricular wall, and decreased cardiomyocyte proliferation in local areas [28]. This is similar to our experimental results. In the cell model, androsterone treatment significantly increased the number of G1-phase cells and decreased S-phase cells. qPCR and WB analysis showed that the expression of cyclins and CDKs was significantly decreased, suggesting cell cycle arrest. These results demonstrated that maternal androgen excess induces decreased fetal cardiomyocytes proliferation through G1–S cell cycle arrest. To our knowledge, we report for the first time that androsterone, a metabolite of testosterone and dihydrotestosterone, exerts an inhibitory effect on cardiomyocyte proliferation through cell cycle arrest. However, whether androsterone is specific for cardiac injury requires further exploration.

RB is the central regulator of cell cycle, which functionally represents a transcriptional corepressor [50]. Hypophosphorylated RB combines with E2F dimers to form complexes, inhibiting transcription of many cell cycle genes involved in the regulation of the G1–S-phase transition [50]. A recent study showed that Down syndrome-associated dual-specificity tyrosine-(Y)-phosphorylation-regulated kinase 1A (DYRK1A), which acts as a negative regulator of hypertrophy in cardiomyocytes, negatively regulated the D-cyclin-mediated RB/E2f-signaling and eventually led to the occurrence of cardiomyopathy [29]. Knockout of RB1 and Meis2 in adult cardiomyocytes induced cell cycle reentry and improved function of cardiac repair after myocardial infarction [51]. These demonstrated that RB could regulate proliferation and differentiation of cardiomyocytes during cardiac development and repair. Our results showed that the phosphorylation of RB was significantly decreased in H9c2 cells treated with androsterone, indicating that androsterone treatment leads to abnormal cardiomyocyte proliferation by decreasing RB phosphorylation and causing G1–S checkpoint transition arrest.

In conclusion, our study demonstrated that in utero exposure to DHT, a direct effect of androsterone on cardiomyocyte cell cycle arrest leads to a decrease in cardiomyocyte proliferation in the embryonic period, which may be related to the changes in cardiac structure and function in adulthood. First, this study reveals the direct damage of androgen on cardiomyocytes in the intrauterine hyperandrogenism environment. Second, it also reminds us to pay attention to the changes in sex hormone levels during pregnancy, especially in patients with PCOS and other diseases that can cause androgen elevation. Finally, since intrauterine exposure to hyperandrogenism is associated with long-lasting cardiac damage in the offspring, we should focus on the follow-up and early intervention of cardiac structure and function in these offspring exposed to hyperandrogenism in utero.

## Limitation

One of the limitations of our study is that the link between androsterone and cell cycle arrest is not clarified. It has been proposed that androsterone and androgen receptors (AR) have weak binding ability [52]. AR belongs to the steroid receptor subfamily of nuclear receptors and plays important roles in the development and maintenance of the reproductive, musculoskeletal, cardiovascular, immune, nervous, and hematopoietic systems [53]. AR, as a nuclear transcription factor, can bind to the promoter region of target genes and induce their expression [53–55]. In addition, AR can also act outside the nucleus, interacting with members of the mitogen-activated protein kinase (MAPK) intracellular pathway to alter their activity [54, 55]. Another limitation is that it is not clear whether there is a molecular basis for the development of cardiac hypertrophy in the adult offspring. One study showed that intrauterine exposure to hyperandrogenism increased the expression of Mef2c in neonatal myocardial tissue [35]. However, further studies are needed to clarify the connection between the elevated genes associated with cardiac hypertrophy in the neonatal period and compromised cardiac function in adulthood, as well as the potential therapeutic approaches. Finally, it is worthy to take concentration on whether the effects of maternal androgen excess on the cardiac health of offspring are in a sex manner.

## Data Availability

The data have not been previously reported and are not under consideration for publication elsewhere. All the raw data are available from the corresponding author on reasonable request.

## Abbreviations

DHT: Dihydrotestosterone
qPCR: Quantitative real-time PCR
RB: Retinoblastoma
pRB: Phosphorylated RB
CDK2: Cyclin-dependent kinase 2
CDK4: Cyclin-dependent kinase 4
CCND1: Cyclin D1
DOHaD: Developmental Origins of Health and Disease
PCOS: Polycystic ovary syndrome
T: Testosterone
H&E: Hematoxylin and eosin
WGA: FITC-conjugated wheat germ agglutinin
BSA: Bovine serum albumin
DAB: 3,3′-Diaminobenzidine
ELISA: Enzyme-linked immunosorbent assay
TUNEL: Terminal deoxynucleotidyl transferase-mediated deoxyuridine triphosphate nick-end labeling
LVPW: Left-ventricular posterior wall thickness
LVID: Left-ventricular internal dimension
IVS: Interventricular septum
LVEF: Left-ventricular ejection fraction
CCNE1: Cyclin E1
CCNE2: Cyclin E2
CCNB1: Cyclin B1
AURKB: Aurora kinase B
β-actin: Actin beta
Nppa: Natriuretic peptide A
Nppb: Natriuretic peptide B
Myh6: Myosin heavy chain 6
Myh7: Myosin heavy chain 7
Acta1: Actin alpha 1, skeletal muscle
Gata4: GATA binding protein 4
Col1a1: Collagen type I alpha 1 chain
Col3a1: Collagen type III alpha 1 chain
Tgfβ1: Transforming growth factor beta 1
Gapdh: Glyceraldehyde-3-phosphate dehydrogenase
EDTA: Ethylenediaminetetraacetic acid
LV: Left ventricular
